# Formulation and *In-vitro* Characterization of Sustained Release Matrix Type Ocular Timolol Maleate Mini-Tablet 

**Published:** 2014

**Authors:** Seyed Alireza Mortazavi, Zahra Jafariazar, Yasaman Ghadjahani, Hoda Mahmoodi, Farzaneh Mehtarpour

**Affiliations:** a *Department of Pharmaceutics, School of Pharmacy, Shahid Beheshti University of Medical Sciences, Tehran, Iran*; b *Department of Pharmaceutics, Pharmaceutical Sciences Branch, Islamic Azad University, Tehran, Iran*

**Keywords:** Ocular drug delivery, Mini-tablet, Timolol maleate, Sustained release, Cellulose derivative, Carbopol 971P, Kinetics models

## Abstract

The purpose of this study was preparation and evaluation of sustained release matrix type ocular mini-tablets of timolol maleate, as a potential formulation for the treatment of glaucoma.

Following the initial studies on timolol maleate powder, it was formulated into ocular mini-tablets. The polymers investigated in this study included cellulose derivatives (HEC, CMC, EC) and Carbopol 971P. Mannitol was used as the solubilizing agent and magnesium stearate as the lubricant. Mini-tablets were prepared by through mixing of the ingredients, followed by direct compression. All the prepared formulations were evaluated in terms of physicochemical tests, including uniformity of weight, thickness, crushing strength, friability and *in-vitro* drug release.

Four groups of formulations were prepared. The presence of different amounts of cellulose derivatives or Carbopol 971P, alone, was studied in group A formulations. In group B formulations, the effect of adding Carbopol 971P alongside different cellulose derivatives was investigated. Group C formulations were made by including mannitol as the solubilizing agent, alongside Carbopol 971P and a cellulose derivative. In group D formulations, mini-tablets were made using Carbopol 971P, alongside two different cellulose derivative. The selected formulation (C_1_) contained ethyl cellulose, Carbopol 971P, mannitol and magnesium stearate, which showed almost 100% drug release over 5 h. Based on kinetic studies, this formulation was found to best fit the zero-order model of drug release. However, the Higuchi and Hixson -Crowell models also showed a good fit. Hence, overall, formulation C_1_ was chosen as the best formulation.

## Introduction

Timolol maleate is a non-selective beta-adrenergic receptor antagonist indicated for treating glaucoma. Glaucoma is a disease in which the optic nerve is damaged, leading to progressive, irreversible loss of vision and topical beta-blockers are often used as a front line drugs for the treatment of glaucoma (1).

Timolol maleate’s chemical name is (-)-l-(tert-butylamino)-3- [(4-morpholino-l, 2, 5-thiadiazol-3-yl) oxy]-2-propanol maleate (1:1) (salt). Timolol maleate has a molecular weight of 432.50. It is a white, odorless, crystalline powder which is soluble in water, methanol, and alcohol (1).

Novel ocular drug delivery systems including hydrogels, drug-loaded soft contact lenses, ocular inserts, ocular mini-tablets, nanoparticles and liposomal drug delivery systems are capable of producing an extended therapeutic effect, compared with the conventional and popular eye drop dosage form (1-4). Eye drops are easy to use, but the bioavailability of the aqueous eye drop is low and only a very small fraction (1.2%) of the instilled dose is available to the target tissues due to the inadequate concentration and insufficient residence time. This is due to the blinking reflex, lacrimation and rapid drainage. Other ophthalmic dosage forms, and in particular novel drug delivery systems, are likely to be more beneficial, and could help to reduce the problem (1-4). In previous studies, ophthalmic mini-tablets have been developed and optimized, showing sustained drug release properties (1-6). Mini-tablets are amongst the novel ocular drug delivery systems, which have been proposed and studied in recent years. In a study, ocular mini-tablets of Diclofenac sodium were prepared and their drug release profile were investigated using three different *in-vitro* methods (static method, paddle method and rotating vial method). The rotating vial method was found to be the best match for *in-vivo* results, showing particularly high *in-vitro/in-vivo* correlation (5). In another study, ocular delivery of Timolol maleate was studied by inserting a controlled-release device, and it was found that this device may offer distinct advantages over the administration of eye drops. They found that the use of ophthalmic insert in rabbit’s eye, could produce a controlled release profile of Timolol Maleate. This insert was made by spraying an aqueous dispersion of acrylic copolymers over a template, producing a thin, rate-controlling membrane. The results also pointed out to the potential validity of coated mini-tablets as simple systems for controlled ocular delivery of Timolol maleate (6).

In a separate study, ophthalmic mini-tablets were made using the natural polymer *Sterculia foetida gum*. In this study, the natural gum and its’ derivatives were used as the gel forming agent, in order to formulate a rate-controlling matrix. This ophthalmic formulation was considered to be capable of improving the bioavailability of ocular drugs (7).

The present study was conducted in an attempt to use a new approach of combining cellulose derivatives and Carbopol 971P, in order to formulate a controlled release mini-tablet matrix, as a potential device for ophthalmic delivery of timolol maleate. The mini-tablets prepared in this study were characterized in terms of uniformity of weight, friability, crushing strength, *in-vitro* drug release and kinetics of drug release.

## Experimental

*Materials*

Ethyl cellulose (EC, 100 cps), hydroxyl ethyl cellulose (HEC, 300 cps) and Carbopol 971P were all purchased from Acros Co.(Geel, Belgium). Magnesium stearate was obtained from JRS Pharma (Madrid, Spain). Timolol maleate was gifted by Sina Daru (Tehran, Iran). Mannitol and sodium chloride were supplied by the Merck Chemical Co. (Darmstadt, Germany). 

*Preparation of mini-tablets*

Table 1 presents the composition of four groups of mini-tablets prepared in this study. Timolol maleate (0.5 mg) and magnesium stearate (0.06 mg) were used in all the prepared formulations. Based on previous studies, the method used for the preparation of mini-tablets was direct compression (8). For this purpose, the formulation ingredients were mixed homogeneously, using pestle and mortar. The powder mixtures were then separately compressed into 3 mm convex mini-tablets, weighing 7 mg each, using an eccentric single punch tablet press (Korsch-Eko model, Berlin, Germany). 

Group A formulations were made using individual polymers. Group B formulations were prepared by adding a cellulose derivative alongside Carbopol 971P, in order to prepare the mini-tablets. Group C formulations were prepared by including mannitol (as a solubilizing agent), besides Carbopol 971P and a cellulose derivative. Finally, group D formulations were made by combining three polymers, with all having Carbopol 971P and two different cellulose derivative. 

**Table 1:** Composition of different Timolol maleate ocular mini-tablet formulations prepared in this study. The final weight of each tablet was 7 mg

**Ingredient**	**Group A (mg)**				**Group B (mg)**			**Group C (mg)**			**Group D (mg)**		
	**A**_1_	**A**_2_	**A**_3_	**A**_4_	**B**_1_	**B**_2_	**B**_3_	**C**_1_	**C**_2_	**C**_3_	**D**_1_	**D**_2_	**D**_3_
**Timolol maleate**	0.5	0.5	0.5	0.5	0.5	0.5	0.5	*0.5*	0.5	0.5	0.5	0.5	0.5
**Magnesium stearate**	0.06	0.06	0.06	0.06	0.06	0.06	0.06	0.06	0.06	0.06	0.06	0.06	0.06
**Carbopol 971P**	6.44	—	—	—	0.32	0.32	0.32	0.20	0.20	0.20	0.20	0.20	0.20
**Mannitol**	—	—	—	—	—	—	—	1.40	1.40	1.40	—	—	—
**EC**	—	6.44	—	—	6.12	—	—	4.84	—	—	3.12	3.12	—
**CMC**	—	—	6.44	—	—	6.12	—	—	—	4.84	3.12	—	3.12
**HEC**	—	—	—	6.44	—	—	6.12	—	4.84	—	—	3.12	3.12

*Characterization of the prepared mini-tablet formulations*

The prepared mini-tablets underwent various physiochemical tests, including the uniformity of weight, crushing strength, friability and *in-vitro* drug release, as follows:

*Uniformity of weight*

The uniformity of weight of the prepared mini-tablets was determined through weighing 10 tablets individually, using an electrical balance with an accuracy of 0.0001 g (Mettler, Germany), and then calculating the average weight and standard deviation (2).

*Crushing strength (Hardness)*

Mini-tablets require a certain amount of strength (hardness) and resistance to friability, to withstand mechanical shocks of handling during manufacturing, packaging and transportation. The crushing strength of the mini-tablets was determined using a hardness tester (Model TBH28, Erweka, Germany) (2).

*Friability*

The friability of the prepared mini-tablets was determined by weighing 10 tablets together. Then, the tablets were placed alongside 10 glass beads (with an average diameter of 4 mm) in a pharma-test friabilator (Model S. 48-3cm, Iran), set at a speed of 25 rpm and faced falling shocks for 10 min. After 10 min, the glass beads were removed and tablets were re-weighed in order to determine the percentage of friability, based on the following equation (2).

$\mathrm{\% Loss}=\frac{\mathrm{Initial weig}ht-\mathrm{Final weig}ht}{\mathrm{Initial weig}ht}\times 100$

*Thickness*

The thickness of 10 randomly selected tablets from each formulation was determined in mm using a graduated caliper (2).

*In-vitro drug release*

The release of Timolol maleate was examined using glass vials in an oscillating water bath. Each mini-tablet was accurately weighed and then transferred into a glass vial containing 1 mL of NaCl 0.9%. In order to avoid evaporation, the vials were covered with rubber caps. They were then placed in an oscillating (25 rpm) water bath at 32 ± 1 °C. Throughout the experiment, 300 µL aliquots were withdrawn at 20, 40, 60, 80, 120, 180, 240 and 300 min intervals, and subsequently replaced by an equal volume of NaCl 0.9%. The samples were measured using a UV spectrophotometer (UV-VIS1201, Shimadzu, Japan) at 294 nm (2).

In order to determine the amount of Timolol maleate released from the studied minitablet, a calibration curve of Timolol maleate was constructed in NaCl 0.9%. The absorbance values were measured using a UV spectrophotometer (UV-VIS 1201, Shimadzu, Japan) at 294 nm (9-12). The obtained calibration curve was found to be linear (y=0.02050x -0.00921,$R^{2}=0.9998).$

*Complementary studies on the selected formulation*

The selected formulation underwent additional studies, including the assay of active ingredient and kinetic profile of drug release. The kinetic models (Table 2) investigated in this study included zero order, first order, Higuchi, Korsmeyer-Peppas and Hixson-Crowell models. Excel 2007 (Microsoft, Redsmond, USA) software was used for calculation of the release rate constants (k _x_), with the aid of solver tool. Statistical evaluation of the different properties of formulated minitablets was performed, using the one-way analysis of variance (ANOVA), along with the TuckE post Hoc test. For this purpose SPSS version 20 software was used. A statistical significance was defined at p < 0.05. 

The assay method used was based on that explained in a previous study, using a UV spectrophotometer (UV-VIS 1201, Shimadzu, Japan) at 294 nm (9-12).

**Table 2:** The mathematical models used to investigate the kinetics of drug release in this study.

**Mathematical model**	**Formula**
Zero order	**Q**_t=_ ** k **_0_ ** t**
First order	ln** Q**_t=_ ln** Q**_0+_** k **_1_** t**
Hixson-Crowell	Q0-3Qt3=k HC t
Higuchi	Qt= k Hn2
Korsmeyer – Peppas	**Q**_t/_ ** Q**_∞_ ** = K**_KP_ ** t** n

Qt = total amount of drug dissolved in time; Q0 = initial amount of drug within the tablet; Qt/ Q∞ = fraction of drug released in time t; n= defines the mechanism of release profile based on the Fick’s law.

## Results and Discussion

As stated earlier, the aim of this study was the preparation of a Timolol maleate containing matrix type mini-tablet formulation, as a potential device for ophthalmic drug delivery. In here a new approach of using different hydrophilic and hydrophobic polymers, for the preparation of mini-tablets, was employed. The results obtained have been presented and discussed in the following sections.

*Results of group A ocular mini-tablet formulations*

As stated before, the formulations in group A contained individual polymers. Results showed that formulation A_1_, which contained Carbopol 971P, could not be compressed in to mini-tablets, and hence left out of the study. Formulations A_3_ and A_4 _produced mini-tablets with rather low crushing strength values, being outside the acceptable range of 0.1 - 1.8 KP (2). This could be explained in terms of the low compressibility of the cellulose derivatives present in these formulations. Hence, it seems that cellulose derivatives used in this formulation can not produce enough integrity in order to desirably compress the ingredients present within the mini-tablets. Moreover, these two formulations completely disintegrated within 10min, and as a result were left out of further studies. 

Formulation A_2 _was found to have a crushing strength of above 2KP and a friability value of less than 1%. In addition, a desirable mini-tablet formulation should be thin, in order to have an acceptable patient compliance. The acceptable thickness for mini-tablets is 1mm (2). The thickness of formulation A_2_ mini-tablet was found to be within the acceptable limit. Hence, formulation A_2_ was found to be the only suitable formulation within group A, which could also withstand disintegration. However, the drug release studies showed that formulation A_2_, which contained ethyl cellulose as the retarding polymer, released almost no drug within 5 h. This is presumably due to the high hydrophobic nature of this polymer, preventing any water entrance into the tablets and hence no drug release. 

Overall, based on the results obtained from group A formulations, none were found to be suitable for further studies.

*Results of group B ocular mini-tablet formulations*

As mentioned in a previous study (2), the presence of Carbopol within mini-tablet formulations, as a release controlling agent, could help to control and improve the drug release properties. Since formulations prepared in group A were none of the suitable, in group B formulations it was decided to add Carbopol 971P as a hydrophilic polymer capable of swelling and hence allowing the entrance of water into tablets in order to diffuse out the incorporated drug content. As a result, in group B formulations, Carbopol 971P was used alongside a cellulose derivative (CMC, HEC or EC). The results obtained from these formulations have been shown in Table 3.

The uniformity of weight in all the group B formulations was found to be within the acceptable range of 7-8 mg (2). Hence, all these formulations complied with the acceptable criteria set for this test and the difference observed were not statistically significant (p> 0.05). The thicknesses of all mini-tablets in group B formulations were also within the acceptable limit of 1 mm (2). Regarding the crushing strength values, formulation B_1_ showed the highest value, followed by B_2 _and B_3_. However, the differences between the hardness values were found to be insignificant (p > 0.05, ANOVA). The highest crushing strength in group B formulations was observed in formulation B_1_, since the combination of Carbopol 971P and EC hardens the mini-tablets. 

In terms of the friability, all the three formulations examined produced friabilities above 1%, which is not acceptable and is in excess of the acceptable limit of 1%.

**Figure1:**
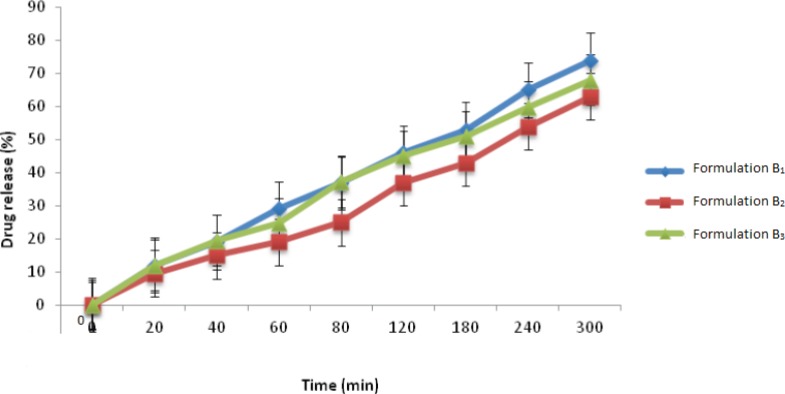
Timolol maleate release profile from group B ocular mini-tablet formulations in NaCl 0.9% at 32±1 ^o^C (n=3, mean±SD).

Finally, when considering the percentage of drug released from this group of formulations (Figure 1), formulation B_1_ released 74% of its drug content within 5 h, which was found to be the greatest amount of drug release between the three formulations investigated in this group. This is probably due to the fact that EC is a highly hydrophobic polymer, which when used alongside the hydrophilic polymer, Carbopol 971P, provides a more porous structure which can help to enhance the entry of water within the tablet matrix and therefore a greater amount of drug release. The other two polymers, namely CMC and HEC, are hydrophilic polymers and when placed in contact with an aqueous medium, absorb water and form a dense and highly viscous gel-like matrix which hinders the exit of drug. Therefore, the amount of drug release from these two formulations was less than formulation B_1__._

Overall, based on the results obtained from group B formulations, none were found to be acceptable in term of all the studies conducted.

**Table 3:** Results of *in-vitro* tests carried out on group B mini-tablet formulations )results presented as mean±standard deviation).

**Formulation**	**Weight (mg)** **(n=10)**	**Crushing strength (KP)** **(n=10)**	**Friability (%)** **(n=1)**	**Cumulative Drug release after 5h (%)** **(n=3)**
B_1_	7.4±0.05	1.16±0.06	1.40	74.01 ±2.5
B_2_	7.4±0.06	1.15±0.05	1.53	63.84 ±3.1
B_3_	7.3±0.03	1.09±0.10	1.01	68.81 ±3.2

*Results of group C ocular mini-tablet formulations*

In this group, the aim was to improve the release profile of drug release by reducing the amount of Carbopol 971P within the formulations and adding mannitol as the solubilizing agent (2). For this purpose different formulations containing Timolol maleate, magnesium stearate, Carbopol 971P, cellulose derivatives and mannitol were prepared. Mannitol is a highly hydrophilic substance and could enhance the in-flow of water from the external medium into the tablet, besides itself being dissolved and hence can increase the rate of drug release from the tablet formulation. The results obtained have been shown in Table 4.

The uniformity of weight in group C formulations was in the normal range of 7-8 mg (7). Formulation C_1 _showed the greatest crushing strength and the lowest friability among the formulations investigated. This could be due to the presence of mannitol, which can presumably increase the hardness as a result of having a good degree of compactibility. Formulations C_2_ and C_3_ had friability values in excess of 1%, making them unsuitable for further studies. The greater friability values of these two formulations could be due to the presence of CMC and HEC alongside mannitol. These polymers have lower compactibilities compared to mannitol and hence lowered the hardness and increased the friabilities of mini-tablets prepared.

The thickness values of all the mini-tablets in group C formulations were within the acceptable limit (2). The statistical analysis of the results observed was not significant (p > 0.05).

**Figure 2:**
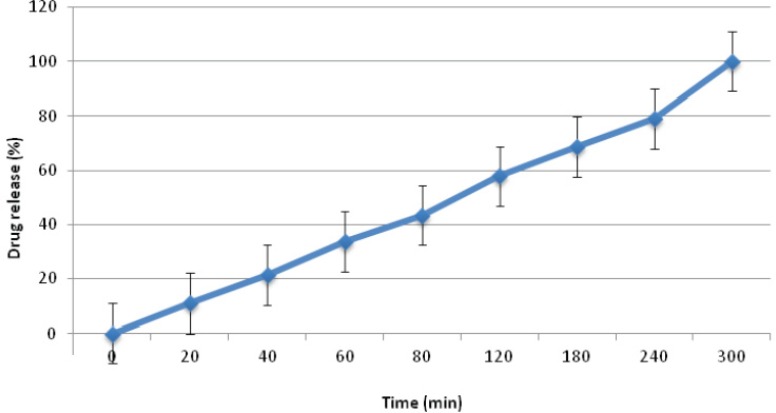
Timolol maleate release profile from formulation C1 ocular mini-tablet formulations in NaCl 0.9% at 32±1 ^o^C (n=3, mean ± SD).

In terms of the profile of drug release, formulation C_1_ showed the most appropriate amount of drug release, which could be due to the presence of mannitol, alongside ethyl cellulose and Carbopol 971P, enhancing the rate of drug release as mentioned before. This formulation was chosen as the selected formulation from this group, because of having suitable properties (Figure 2). Formulation C_2 _and C_3 _released all their drug content within 20 min, due to the presence of HEC in formulation C_2_ and CMC in formulation C_3_ in place of EC. Since HEC and CMC are hydrophilic polymers, they could result in an over-flow of water into the formulation, resulting in over-hydration of the mini-tablets and their disintegration. However, EC is a hydrophobic polymer which can reduce the amount of water uptake compared to CMC and HEC. This could help to balance the amount of water uptake by the mini-tablet formulation and hence the suitable profile of drug release observed. 

**Table 4:** Results of *in-vitro* tests carried out on group C mini-tablet formulations. )results presented as mean±standard deviation).

**Formulation**	**Weight (mg)** **(n=10)**	**Crushing strength (KP)** **(n=10)**	**Friability (%)** **(n=1)**	**Cumulative drug release after 5h (%)** **(n=3)**
C_1_	7.2±0.05	1.59±0.53	0.53	100±0.5
C_2_	7.3±0.01	0.90±0.17	1.11	100 % in 20 min
C_3_	7.4±0.07	0.82±0.18	1.18	100 % in 20 min

*Results of group D ocular mini-tablet formulations*

In group C formulations it was found that the presence of HEC and CMC resulted in quick drug release from the mini-tablets, which is undesirable. Hence, it was decided to omit mannitol from these formulations. As a result, combinations of different cellulose derivatives (HEC, CMC or EC) alongside Carbopol 971P were prepared (2). The results obtained from these formulations (group D) have been shown in Table 5. The weight of mini-tablets prepared in this group was found to be within the acceptable range (2). However, when examining the crushing strength of mini-tablets prepared, formulation D_1 _containing EC and CMC alongside Carbopol 971P, showed the greatest hardness. Statistical analysis of the result showed a significant difference between the crushing strength of formulation D_1_ with the other two formulations (p < 0.05). Based on this finding, it seems that the combination of CMC along with EC can produce mini-tablets with a greater strength.

**Figure 3:**
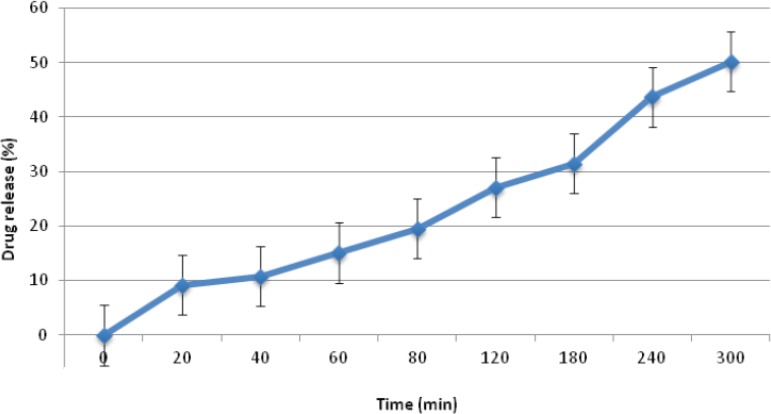
Timolol maleate release profile from formulation D_1_ ocular mini-tablet formulations in NaCl 0.9% at 32±1 ^o^C (n=3, mean±SD).

Regarding the friability values obtained for group D formulations, none were found to be suitable, showing friability values in excess of 1%. This finding seems to be due to the low hardness values of these mini-tablet formulations, making them unsuitable for further studied. Finally, in terms of drug release studies, formulations D_2_ and D_3_ released all their drug content within 20 min and 80 min, respectively. Moreover, formulation D_1_ only managed to release around 50% of it’s drug content after 5 h (Figure 3). Hence, based on the results obtained, none of the formulations prepared in group D were found to be acceptable for further studies.

**Table 5:** Results of *in-vitro* tests carried out on group D mini-tablet formulations. (results presented as mean±standard deviation).

**Formulation**	**Weight (mg)** **(n=10)**	**Crushing strength (KP)** **n=10**	**Friability (%)** **n=1**	**Cumulative drug release after 5h (%)** **n=3**
D_1_	7.6±0. 07	0.82±0.16	1.71	47.91±0.8
D_2_	7.5±0.09	0.75±0.17	1.80	100 % in 80 min
D_3_	7.5±0.35	0.74±0.18	1.90	100% in 20 min

*Complementary studies on the selected formulation *C_1_

Based on the results obtained, formulation C_1_ which had a suitable profile of drug release alongside an acceptable crushing strength and friability values was chosen as the selected formulation among four groups of mini-tablet formulations investigated in this study. This formulation underwent additional studies, namely assay of the active ingredient and the kinetic profile drug release. Results showed that the amount of Timolol maleate present within this formulation was 95 ± 0.5 % (n=3), which was found to be within the acceptable range of 90-110% (9-12). Hence, the assay method used (uv-visible spectroscopy) seems to be suitable for this purpose. Next, the kinetic studies were carried out on formulation C_1_ in order to determine the most suitable mathematical method which describes the profile of drug release from this formulation (18). The mathematical models investigated included first order, zero-order, Hixson-Crowell, Higuchi model and Korsmeyer- Peppas. Results (Table 6) showed that the profile of drug release from formulation C_1_, best fits the zero order kinetics. This means that the drug released from formulation C_1_, follows a constant rate of release. However, the Higuchi and Hixson -Crowell models also showed a good fit. Based on the n-value obtained from the Korsmeyer- Peppas equation (n = 0.9227), again fitting the profile of drug release from formulation C_1_ to zero order kinetics, can be justified (18). This would mean that formulation C_1_ is capable of releasing its’ drug content in a controlled manner, and hence is expected to provide a steady drug concentration in the eye for an extended period of time. 

**Table 6:** Release rate constants and correlation coefficients obtained after fitting various mathematical models into the release profile of formulation C_1_

**Mathematical model**	**K**	$R^{2}$	n
Zero order	17.9460	0.9882	-
First order	0.4783	0.8630	-
Hixson-Crowell	0.4907	0.9551	-
Higuchi	53.6680	0.98882	-
Korsmeyer-Peppas	21.6421	0.9551	0.9227

## Conclusion

In conclusion, it seems that the use of the hydrophobic polymer ethyl cellulose in combination with the hydrophilic polymer Carbopol 971P, alongside mannitol, could provide a good template for the preparation of a desirable matrix type ocular Timolol maleate mini-tablet. This combination could provide a controlled profile of drug release, which can presumably help to manage the disease much better than the conventional Timolol maleate eye drop formulation.
